# Impact of traveling on transmission trends of human monkeypox disease: worldwide data based observational analysis

**DOI:** 10.3389/fpubh.2023.1029215

**Published:** 2023-06-14

**Authors:** Sultan Ayoub Meo, Thamir Al-Khlaiwi, Fawzi Fahad Al Jassir, Anusha Sultan Meo

**Affiliations:** ^1^Department of Physiology, College of Medicine, King Saud University, Riyadh, Saudi Arabia; ^2^Department of Orthopedics, College of Medicine, King Saud University, Riyadh, Saudi Arabia; ^3^College of Medicine, King Saud University, Riyadh, Saudi Arabia

**Keywords:** human monkeypox, monkeypox, travelers, transmission, spread

## Abstract

**Background:**

Human monkeypox is an emerging viral zoonotic disease caused by a monkeypox virus (MPXV). This year since early May 2022, the virus swiftly spread involved 94 countries, and 41,358 people, and has developed a highly challenging and threatening situation worldwide. This study aimed to investigate the impact of traveling on the transmission of human monkeypox disease and comprehend the link between monkeypox exported cases in the context of the global outbreak.

**Methods:**

In this study, we identified data from two leading health organizations, the World Health Organization (WHO), and the Centers for Disease Control Prevention (CDC), as well as 40 documents that were identified through the search engines Web of Science, Pub-Med, Medline, EMBASE, Scopus, and Google Scholar using the keywords “monkeypox,” “human monkeypox,” “imported”’, “exportation” “travelers,” and “prevalence.” Finally, two international organizations WHO, and CDC, and out of 40 documents, 10 (25.0%) were included in the analysis, and the remaining 30 (75.0%) documents were excluded. The studies originated from the United Kingdom, the United States of America, Singapore, Israel, the Republic of Korea, Taiwan, and India. The data on transmission trends and human monkeypox was recorded and analyzed.

**Results:**

The epidemiological data for exported monkeypox cases were analyzed jointly for understanding the transmission trends of exportations and the geographic context of the monkeypox outbreak. Ten people had a travel history, six had a travel history from Nigeria to the United Kingdom (2), Nigeria to the United States of America (2), Nigeria to Singapore (1) and Nigeria to Israel (1). Moreover, from Germany to Taiwan (1), Germany to the Republic of Korea (1) and the United Arab Emirates to India (2). Among these 10 people, all travelers were male (100%), with age ranges of 20–38 years, seven people (70%) developed clinical symptoms before the start of travel, three people (30%) developed symptoms 2–6 days after the travel, and one person (10%) developed clinical symptoms in the flight during the journey.

**Conclusion:**

The study findings conclude that traveling can cause the spread of human monkeypox disease in various countries. The findings support the hypothesis that virus sources can travel and spread the disease from person to person and from region to region. The international health authorities must implement global preventive policies to control the burden of the disease both at regional and international levels.

## 1. Introduction

Human monkeypox, commonly known as monkeypox disease is caused by a monkeypox virus (MPXV), which is a zoonotic infection (1, 2). The MPXV belongs to the “genus *Orthopoxvirus*, subfamily *Chordopoxvirinae* and family *Poxviridae*.” The monkeypox family viruses have numerous pathogenic characteristics and infect humans (3–5).

The monkeypox virus was found for the first time in 1958 in monkeys which were housed in the research institute in Copenhagen, Denmark, hence the disease acquired the name monkeypox (6). About 12 years later, in September 1970, the MPXV was first time identified in humans when a 09-months, old infant was admitted to the hospital in the Democratic Republic of Congo (7). In October 1970 and May 1971, six cases of human MPXV were identified in “Liberia, Nigeria, and Sierra Leone” and 10 cases were reported between the years 1971 and 1978 (8–10).

However, in the new millennium, in the year 2003, the human monkeypox disease was for the first time transmitted from the endemic African region to non-endemic nations in the United States of America (11). In 2003, 47 confirmed and suspected cases of monkeypox infection were reported in six states in America. These states include Indiana, Kansas, Illinois, Ohio, Wisconsin, and Missouri. The people who suffered from monkeypox infection had a history of contact with pet prairie dogs. The pets contracted the infection after being housed near the small mammals imported from Ghana, which led to the occurrence of the first case of human monkeypox infection outside Africa (12, 13). It is important to understand that the outbreak of monkeypox in 2003 in the US reflects the virus’s capability of transmission outside the place of origination, Central Africa, and from region to region (13).

During one year of period, from January 1, 2022, to January 5, 2022, the virus has adopted multiple paths of transmission and has swiftly spread from endemic to non-endemic regions, involving 110 countries, infecting 84,318 people: 1,191 cases from 07 endemic African countries and 83,127 cases in 103 non-endemic countries in the Europe, America, Australia, and Asian continents (14). The disease also caused 74 deaths in 17 countries worldwide (14). The transmission trends of human monkeypox disease have rapidly changed, and the virus is not only limited to close contact with humans. Moreover, there is debate in the science community about the association of the human monkeypox virus with travel. While the rapid appearance of monkeypox cases across the world demands comprehensive epidemiological investigations to understand the link between travel and the spread of monkeypox cases. Therefore, this study aimed to investigate the association of traveling with the transmission trends of human monkeypox disease and the context of the outbreak.

## 2. Research methodology

This study was performed in the Department of Physiology, College of Medicine, King Saud University, during the period July–August, 2022.

### 2.1. Data collection & study characteristics

This study explores the impact of traveling on the transmission trends of human monkeypox disease. The literature was searched from global reputable publishing organizations and websites. In this study, Initially, two leading health organizations, the “World Health Organization (WHO) (1), and the Centers for Disease Control Prevention (CDC)” (2, 14), and 40 articles were identified through search engines, Web of Science (15), Pub-Med (16), Medline, EMBASE, Scopus, Google scholar using the keywords “monkeypox, human monkeypox, imported, exported, travelers, prevalence.” Finally, two international organizations “The World Health Organization (WHO) (1), and the Centers for Disease Control Prevention (CDC)” (2), and out of 40 documents, 10 (25.0%) were included in the analysis, and the remaining 30 (75.0%) documents were excluded. The data on transmission trends and human monkeypox were recorded and analyzed. The 10 publications and reports which were finally included in the analysis were recorded all around the world during the period from January 2017 to August 2022. These studies originated from the United States of America, the United Kingdom, Singapore, Israel, the Republic of Korea, Taiwan, and India.

The data on travel and its association with the spread of monkeypox cases were recorded and analyzed based on community-based studies with a travel history and confirmed monkeypox disease. The appropriateness of documents was examined by the title and abstract of the reports. Once a document was shortlisted, relevant findings were recorded by an investigator and verified by another investigator. Inclusion and exclusion criteria for selecting the studies were standardized, and documents must have been published by leading health organizations WHO, and CDC, and in peer-reviewed, “PubMed and Web of Science indexed journals” on human monkeypox that investigated the impact of the travel and spread of the human monkeypox cases. However, studies published in journals which were not indexed in ‘Pub-Med’ and ‘Web of Science’ were excluded. All the selected 10 articles were carefully examined. The required data were recorded, including the authors’ name, publication year, location, sample size, travel history, study outcomes, and monkeypox allied detailed information.

### 2.2. Ethical approval and statistical analysis

The data was obtained from publicly available databases hence ethical approval was not required. The data were carefully reviewed, and findings were recorded and analyzed. The analysis was based on each study’s findings. The number and percentages were calculated and presented.

## 3. Results

**Figure 1:**
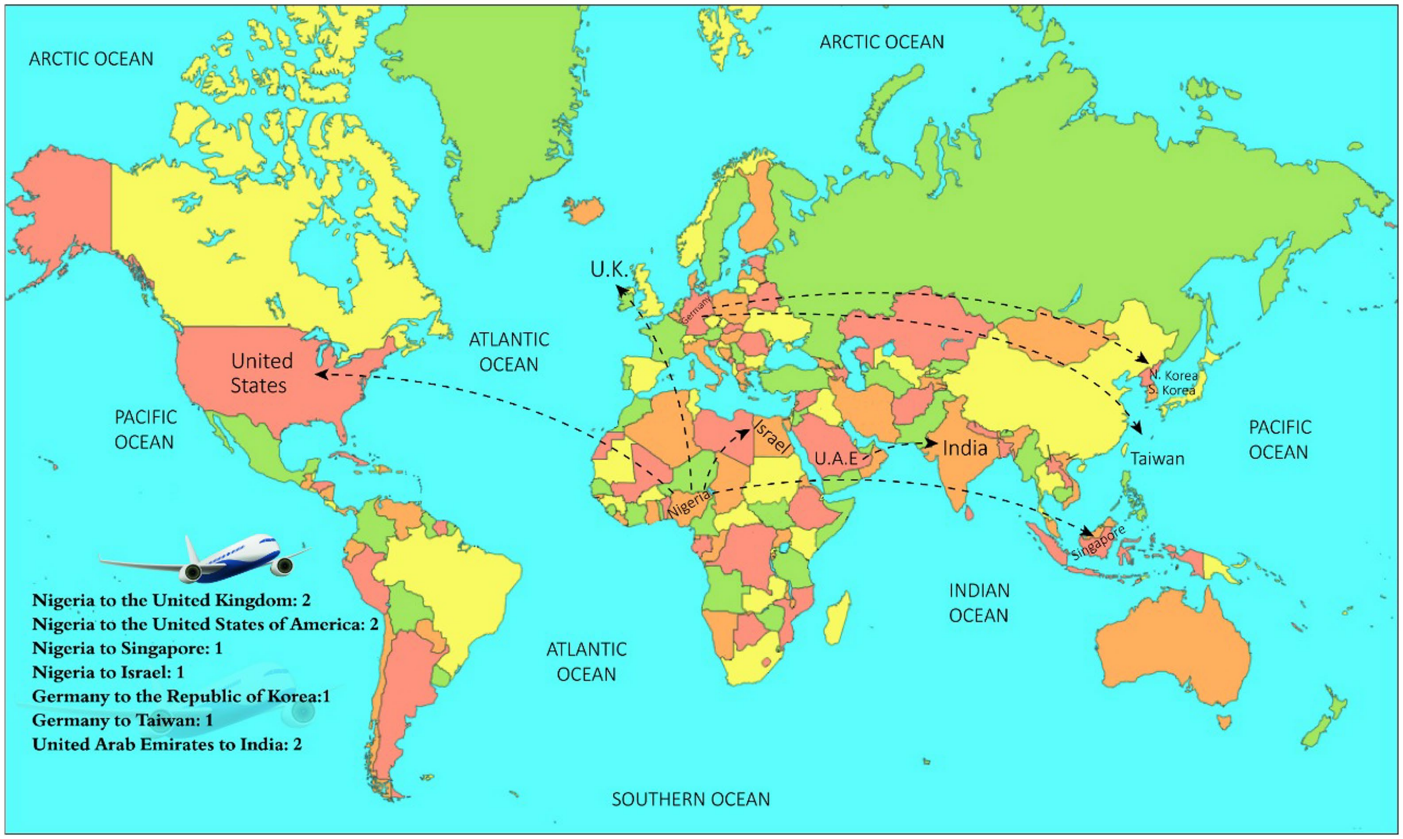
Traveling and exportation of monkeypox cases.

In this study, we identified 40 articles from different web search engines, and after an in-depth review, finally, data from 2 international organizations and 8 studies with data of 10 cases were included. These 8 studies originated from multiple countries and presented monkeypox cases worldwide. These studies originated from the United Kingdom, Singapore, Israel, the United States of America, the Republic of Korea, Taiwan, and India (Table 1; Figure 1).

**Table 1:** Traveling history, demographic data, and appearance of clinical features in travelers from various countries.

Study	Type of study	Reporting country	Traveler’s age and gender	Travelling history	Clinical symptoms onset before or after the travel	Monkeypox case confirmation
Vaughan et al. (17)	Article	United Kingdom	32-year male Nigerian Naval officer	Traveled from Nigeria to the UK, on September 2, 2018	Before travel on September 1, 2018	Confirmed case of monkeypox on September 7, 2018.
Vaughan et al. (17)	Article	United Kingdom	36 years old male	Traveled from Nigeria to the UK, flight Via Paris-France on September 4, 2018	Before travelling on September 3, 2018	Confirmed case of monkeypox on September 11, 2018.
Yong et al. (18)	Article	Singapore	38 years old male	Traveled from Nigeria to Singapore on April 28, 2019	After 2 days of travel on April 30, 2019	Confirmed case of monkeypox on May 8, 2019.
Erez et al. (19)	Article	Israel	38 years old male	Traveled from Nigeria to Israel on September 23, 2018,	After 6 days of travel, on Sept 29 patient developed itchy skin lesions, fever, and chills.	Confirmed case of monkeypox on October 7, 2018.
Rao et al. (20)	Article	Delas, USA	Middle-aged male	June 25, 2021, arrived in Nigeria and returned from Nigeria to Atlanta, the USA on July 8, 2021, and Atlanta to Delas USA on July 9, 2021	Before 1 week, returned from Nigeria to the USA. On June 30, 2021, the patient had a cough, vomiting, fever & fatigue.	Confirmed case of monkeypox July 13, 2021
Costello et al. (21)	Case report	Maryland, USA	28 years old male in the USA	Traveled from Lagos Nigeria to Maryland, USA November 21, 2021.	On a flight from Lagos, the patient developed a burning sensation, on skin, discrete vesicles	Confirmed case of monkeypox
Jang et al. (22)	Article	Republic of Korea	34 years old male	Traveled from Germany to Korea on June 21, 2022, and stayed in Germany from June 1 to 21, 2022.	Before arrival in Korea, he had a headache on June 19, 2022, and on arrival in Korea developed skin lesions.	Confirmed case of monkeypox on 5th day in the hospital, on June 25, 2022
Yang et al. (23)	Article	Taiwan	20-year-old male student	Traveled from Germany to Taiwan, on June 16, 2022	After 4 days, on June 20, 2022, developed a fever, sore throat, skin rashes, lymph node swelling	Confirmed case of monkeypox on June 24, 2022
Yadav et al. (24)	Letter	India	35-year, male, Engineer	Traveled from Sharjah, United Arab Emirates to Kerala, India, on July 12, 2022.	Before travelling to India, on July 5, 2022, he developed symptoms	Confirmed case of monkeypox on July 13, 2022
Yadav et al. (24)	Letter	India	31-year, male, bus driver	Traveled from Dubai, United Arab Emirates to Kerala, India 13 July 2022	Before travelling to India, on July 8, 2022, developed symptoms	Confirmed case of monkeypox on July 16, 2022

**Figure 2:**
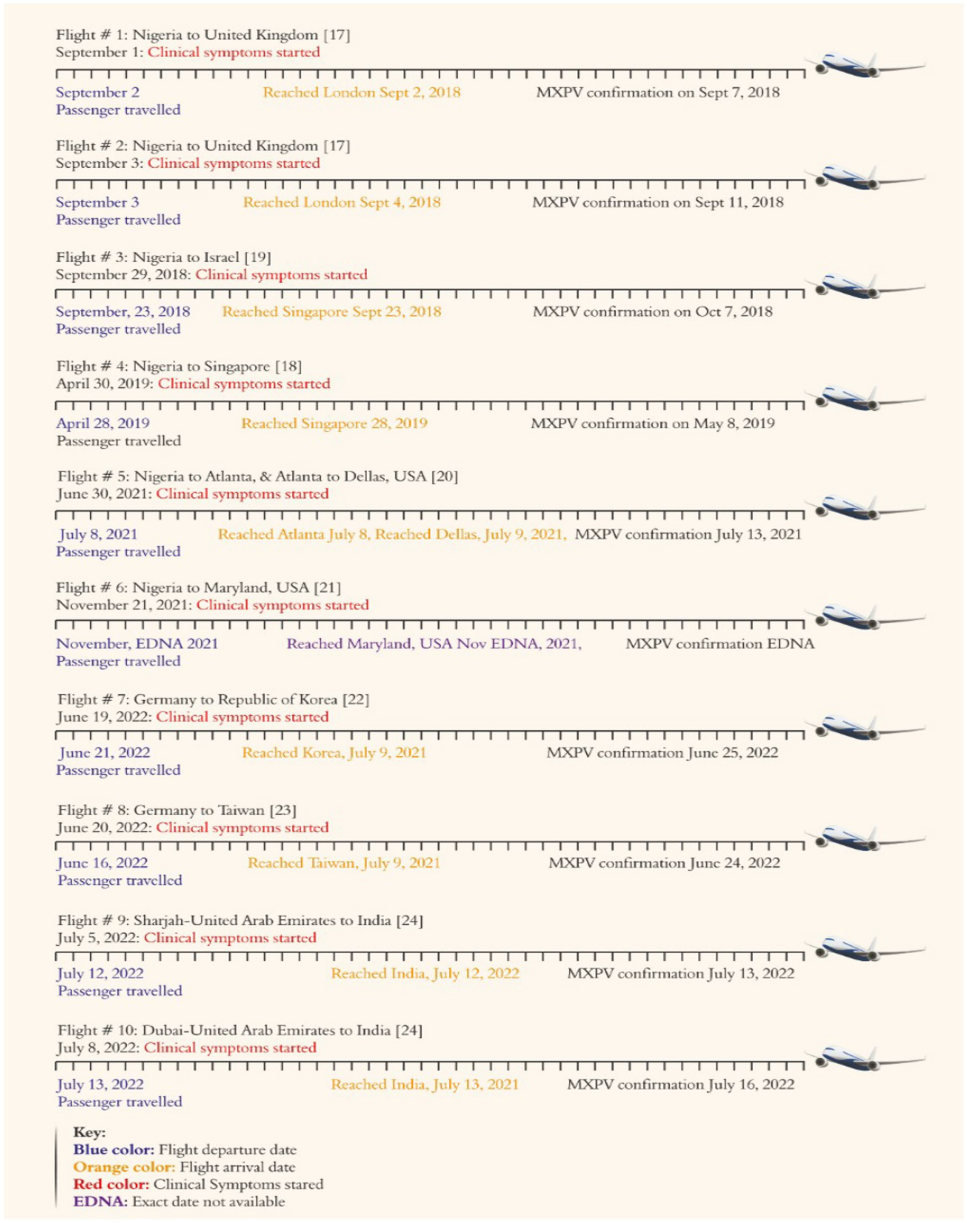
Traveling history and demographic data of travelers who export the monkeypox virus.

Table 1 demonstrates the demographic and epidemiological data for people who traveled from Nigeria to multiple countries worldwide. The present study identified that the six patients had a travel history from Nigeria to the United Kingdom (2), Nigeria to the United States of America (2), Nigeria to Singapore (01) and Nigeria to Israel (01). Moreover, from Germany to Taiwan (01), and Germany to the Republic of Korea (01), 02 patients had a travel history from the United Arab Emirates to India (Table 1; Figures 1, 2). Among these 10 patients, all travelers were male (100%), with age ranges of 20–38 years. The results reveal that seven people (70%) developed clinical symptoms before the start of travel, three (30%) developed symptoms 2–6 days after travel, and one person (10%) developed clinical symptoms in the flight during the journey (Table 1; Figure 2).

The results revealed that traveling was associated with the spread of human monkeypox disease from one region to another. The findings support the hypothesis that virus sources can travel and spread the disease from person to person and country to country (Table 1). For further description, the travel-allied findings retrieved from the global literature are summarized below.

### 3.1. Travel history and demographic data of the travelers

#### 3.1.1. Case 1: travel history – Nigeria to the United Kingdom

A 32-year-old male Nigerian naval officer traveled from Abuja, Nigeria to London, and London to a military base in the southwest of England dated September 2, 2018. The case was presented to the general practitioner dated 3 September with fever, skin rashes and lymphadenopathy. The clinical symptoms developed the day before leaving Nigeria. On 6 September 2018, skin rashes appeared on the face and arms, and monkeypox virus DNA was detected on September 7, 2018 (17).

#### 3.1.2. Case 2: travel history – Nigeria to the United Kingdom

A 36-year-old Nigerian, a UK-based male resident, returned after spending a 22-day holiday in Nigeria on 4 September 2018. On September 6, the patient presented to the hospital with a fever, itchy maculopapular rashes, and lymphadenopathy. The skin rashes on the face and palms of the hands appeared before departing from Nigeria dated September 3, 2018. The monkeypox DNA was confirmed by multiple molecular assays on 11 September 2018. The patient had a contact history with a person in a family gathering who had monkeypox-like clinical features and consumption of bush meat during his visit to Nigeria (17).

#### 3.1.3. Case 3: travel history – Nigeria to Singapore

A 38-year-old man traveled from Nigeria to Singapore on April 28, 2018. The patient held an administrative job and had no history of contact with rodents or people with pox-like illnesses. After arriving in Singapore, the patient developed fever, chills, muscle and body pain on April 30, and vesicular rashes on the face on May 1. He sought medical attention on May 7; the physician notified him of a suspected monkeypox case. The patient had a fever, and multiple pustular lesions over the face, trunk, limbs, palms, and soles with cervical and inguinal lymphadenopathy. The monkeypox diagnosis was made on May 8, 2019 (18).

#### 3.1.4. Case 4: travel history from Nigeria to Israel

A 38-year-old man, resident of Israel, worked at a desk job for the previous 10 years in Nigeria. He traveled from Port Harcourt, Rivers State, Nigeria to Israel on September 23, 2018. After 6 days of his travel, on September 29, the patient noticed 2 itchy lesions on the body, fever, and chills. From October 1 to October 4, 2018, patient developed maculopapular rashes on the face, neck, trunk, lower and upper extremities, palms, and soles and bilateral enlarged and tender lymph nodes in the groin region. The oral examination revealed bilateral tonsillar enlargement and ulcers in the oral cavity. The pustule sample was processed for PCR analysis which confirmed the monkeypox case (19).

#### 3.1.5. Case 5: travel history – Nigeria to Dallas, Texas, USA

A middle-aged, male arrived in Nigeria dated June 25, 2021, stayed in three different urban places, attended a social gathering, and returned from Nigeria to Atlanta, USA on July 8, 2021. After a brief layover at Atlanta airport, he took a domestic flight and reached Dallas, USA on July 9, 2021. On July 8, before boarding, the patient developed a purulent skin rash, after a brief layover. The next day, the skin rash worsened and was visible on the face, and other parts of the body. The patient also developed cough, vomiting, diarrhea, fever, and fatigue, all characteristic signs, and symptoms of the monkeypox prodrome. On July 13, 2021, the real-time polymerase chain reaction (PCR) confirmed the West African clade monkeypox virus. This was the first travel-associated monkeypox case in the United States since the 2017 outbreak in Nigeria (20).

#### 3.1.6. Case 6: travel history – Nigeria to Maryland, USA

A 28-year-old man had traveled on a flight from Lagos, Nigeria to Maryland, United States dated November 21, 2021. While in Nigeria, he visited relatives and stayed in a hotel with no contact with animals. During his flight from Lagos, he noticed a burning sensation on his skin, followed by the development of discrete vesicles on his forehead, and nose, which spread to his arms, trunk, and inner thighs, without fever, chills, or headache. This case of monkeypox in a traveler returning to the United States from Nigeria demonstrates that monkeypox is a travel-related disease (21).

#### 3.1.7. Case 7: travel history – Germany to the Republic of Korea

A 34-years old Korean man arrived from Germany to the Republic of Korea on June 21, 2022. He stayed in Germany from June 1–21, 2022. He had headache 3 days before traveling to Korea, and on the day of arrival, he found skin lesions. He also learned that his friend in Germany had undergone a diagnostic test for monkeypox at a German hospital. The patient informed the health authorities about their medical condition and contact history with a suspected person having monkeypox-like symptoms. The authorities confirmed a case of monkeypox on the 5th day in the hospital, on June 25, 2022. This is the first case of monkeypox in the Republic of Korea after returning from Europe (22).

#### 3.1.8. Case 8: travel history – Germany to Taiwan

A 20-year-old young male student went for studies in Germany in January 2022 and on June 16, 2022, returned to Taiwan. After 4 days, he developed a fever, sore throat, muscle pain, lymph node swelling and skin rashes. On June 24, 2022, Taiwan CDC confirmed that this patient had a case of monkeypox virus infection (23).

#### 3.1.9. Case 9: travel history – Sharja UAE to India

A 35-year, old male, engineer traveled on 12 July 2022 from Sharjah, UAE to Kerala state India. He developed fever and myalgia on 5 July 2022, and on the next day, he had multiple vesicular rashes in the oral cavity and lips and other parts of the body. He also had maculopapular rashes on both hands. He visited a private hospital in Sharjah on 9 and 11 July 2022 (24).

#### 3.1.10. Case 10: travel history – Dubai UAE to India

A 31-year-old male school bus driver in Dubai, UAE, traveled from Dubai to Kerala, India on dated 13 July 2022. Before the travel, on 8 July 2022, he developed dysuria and genital swelling. On 10 July 2022, he had a fever with chills, headache, backache and myalgia and multiple vesicular rashes on both hands and body parts. The skin lesions progressed and later spread to the face, back, neck and forearm with cervical lymphadenopathy by 15 July 2022. The laboratory diagnosis was conducted using PCR and confirmed the MPXV West African clade (24).

## 4. Discussion

The swift spread of the disease has developed a challenging and threatening situation worldwide (25). More recently, considering the rapid transmission of the monkeypox disease from endemic to non-endemic countries, the World Health Organization declared the disease a global health emergency (26). The present study identified that ten cases who traveled had been exposed to MPXV. Out of these 10 cases, six people had a travel history from Nigeria to the United Kingdom, the United States of America, Singapore, and Israel. Moreover, 02 people traveled from Germany to Taiwan and the Republic of Korea; and 02 persons had a travel history from the United Arab Emirates to India. Among these 10 people, seven people (70%) developed clinical symptoms before the start of travel, three (30%) developed symptoms 2–6 days after travel, and one person (10%) developed clinical symptoms on the flight during the journey (Table 1).

The number of monkeypox cases and the geographical range of the spread of the disease have expanded significantly. Since early May 2022, the rapid occurrence of the disease in various nations worldwide has been unusual and markedly increased which raises international concerns about a possible change in the pattern of spread of the monkeypox disease that may pose a global threat (27). The possible transmission is through respiratory droplets, direct or indirect contact with body fluids, or used material such as cloths, bed sheets, towels, skin lesions of an infected person, and a contaminated patient’s environment has been associated with inter-human transmission (2, 4, 5).

The monkeypox virus DNA lies on surfaces in hospitals and households. The monkeypox virus was also found in air and dust samples that were collected during a bed linen change in rooms used to isolate monkeypox patients (28). MPXV DNA contaminated the environment occupied by the infected symptomatic individuals (28). These observations demonstrate that the transmission trends of the monkeypox virus may have changed from close contact to other routes including through body fluids, respiratory secretions, and polluted personal objects that can contaminate the environment, resulting in the virus spreading among people (28).

The literature highlights the link among patients with a traveling history. The virus transmits through the infected individuals, and contaminated materials including polluted personal objects can contaminate the environment, and result in the virus spreading among people (28). In the present, we identified the 10 cases of monkeypox with a travel history from Nigeria to the United Kingdom, the United States of America, Singapore, and Israel; from Germany to the Republic of Korea, and Taiwan; and from the United Arab Emirates to and India (Table 1).

Vaughan et al., 2018 (17) reported two cases with a travel history from Nigeria to the United Kingdom, both developed clinical symptoms and were confirmed cases of monkeypox. In another study, Yong et al., 2020 (18) reported a monkeypox case of a 38-year-old man who traveled from Nigeria to Singapore. Similarly, Erez et al., 2019 (19) identified a case of monkeypox with a travel history from Nigeria to Israel. All these cases traveled from the endemic region, Nigeria to non-endemic regions the United Kingdom, Singapore, and Israel.

Similarly, Rao et al., 2022 (20) reported a case of monkeypox who had a travel history from Nigeria to Dallas, USA, patient developed purulent skin rashes and was diagnosed as the first travel-allied monkeypox case in the United States. Similarly, Costello et al., 2022 (21) reported a case of a 28-year-old man, who had traveled from Lagos, Nigeria, and arrived in Maryland, United States. During his flight, he developed a burning sensation on skin, and discrete vesicles on his forehead, nose, arms, trunk, and thighs. These travelers visited the monkeypox endemic region, Nigeria and returned to their hometowns in non-endemic countries in the United Kingdom, the United States of America, Singapore, and Israel (Table 1).

More recently, Jang et al., 2022 (22) identified a case of a middle-aged Korean man who traveled from Germany to the Republic of Korea. Three days before traveling to Korea, and on the day of arrival, he found some skin lesions and was diagnosed with a case of monkeypox. This was the first case of monkeypox in the Republic of Korea after returning from Europe (22). Similarly, Yang et al., 2022 (23) reported a 20-year-old young male student who went from Germany to Taiwan. After 4 days of his travel, the student developed a fever, sore throat, muscle pain, lymph node swelling, and skin rashes and health authorities diagnosed a case of monkeypox virus infection (23). Likewise, Yadav et al., 2022 (24) reported two cases of monkeypox, both were traveled from the United Arab Emirates to India.

The literature highlights the increasing incidence of monkeypox cases all around the world. On January 5, 2022, the virus has adopted multiple paths of transmission and has swiftly spread from endemic to non-endemic regions, involving 110 countries, infecting 84,318 people (14). The monkeypox virus has adopted multiple paths of transmission, and it is important to understand the possible pathways of the spread of the disease for the deterrence of the illness. The virus can transmit during traveling through close contact, respiratory droplets, travel environmental conditions or contaminants with the objects during the traveling.

The human monkeypox disease manifested with a variety of dermatologic and systemic clinical manifestations. Majority of the patients presented with a rash, anogenital lesions, and mucosal lesions. The most frequent systemic features are fever, headache, lethargy, myalgia, and lymphadenopathy (29), and neurological manifestation including encephalitis (30).

The most common sites for skin lesions were the anogenital area, trunk, arms, legs, face, palms, and soles. In another study, Adler et al., 2022 (31) described the clinical course of monkeypox in a high-income setting. The authors demonstrated that human monkeypox poses unique challenges, even to nations with well-resourced healthcare systems. The authors found that there was a prolonged upper respiratory tract viral DNA shedding after the skin lesion.

Morgan and colleagues 2022 (32) conducted a study on environmental sampling at the residence of a monkeypox patient in Dallas, Texas. The environmental swab sampling was conducted 15 days after a patient with monkeypox disease left the household. The results indicate an extensive MPXV-WA DNA contamination and viable virus in multiple samples. The findings suggested that porous surfaces, bedding, and clothing might pose more MPXV exposure risk than nonporous surfaces. This evidence shows that travel might become a risk factor to spread the monkeypox disease. The virus can spread through body fluids, respiratory secretions, contact with soiled materials, infected cloths, bed linen, objects, surfaces, and dust particles. This is also a fact that the speed of international transportation combined with the natural progression of the disease, and long incubation periods, increase the risk of monkeypox spreading from region to region while traveling (33). The changes in the ecology and climate, as well as the distribution of disease-transmitting vectors and their hosts, increased the risk of diseases (34). Moreover, the epigenetic modifications may influence viral infectivity and disease severity, and general environmental factors can aid the transmission of the virus based on the lessons we have learned from the COVID-19 era and its environmental effects (35).

## 5. Study strengths and limitations

Despite the limitations, this study has important strengths. First, we highlighted a relevant issue of traveling and the spread of monkeypox cases. Second, the data is based on the appropriate number of patients with a travel history from various regions of the world and transmission of the disease. These strengths assign immense value to the results of this study for the science community, health officials, policymakers and the general public. The limitation of this study is that still, the literature is limited both in scale and in the details required to effectively categorize travel risk.

## 6. Conclusion

The present study identified that ten cases who traveled had been exposed to MPXV. Among them, seven travelers developed the disease before the travel, 3 after the travel and one during the journey. The present study findings and monophyly of exportation cases suggest a likely link with travel allied to monkeypox disease and association with the source for the exported infections from various parts of the world. The monkeypox virus may transmit during traveling through close contact, respiratory droplets, travel environmental conditions or contaminants with the objects during the traveling. The findings suggest that regional and international traveling might be a risk to spreading the monkeypox disease. In this age of increasing travel, country borders have no impact on the movement of pathogens including the MPXV. The international health authorities must implement global preventive policies to control the burden of the disease both at regional and international levels.

## Data availability statement

The raw data supporting the conclusions of this article will be made available by the authors, without undue reservation.

## Ethics statement

Ethical review and approval was not required for the study on human participants in accordance with the local legislation and institutional requirements. Written informed consent for participation was not required for this study in accordance with the national legislation and the institutional requirements.

## Author contributions

SAM: research design and writing and editing the manuscript. TA-K, FA, and ASM: literature review, data checking, and analysis. All authors contributed to the article and approved the submitted version.

## Funding

This study was supported by Deputyship for Research & Innovation, Ministry of Education, Saudi Arabia (IFKSUOR3-4-1).

## Conflict of interest

The authors declare that the research was conducted in the absence of any commercial or financial relationships that could be construed as a potential conflict of interest.

## Publisher’s note

All claims expressed in this article are solely those of the authors and do not necessarily represent those of their affiliated organizations, or those of the publisher, the editors and the reviewers. Any product that may be evaluated in this article, or claim that may be made by its manufacturer, is not guaranteed or endorsed by the publisher.
